# In vitro anticandidal potency of *Syzygium aromaticum* (clove) extracts against vaginal candidiasis

**DOI:** 10.1186/s12906-020-2818-8

**Published:** 2020-01-30

**Authors:** Mohamed Taha Yassin, Ashraf Abdel-Fattah Mostafa, Abdulaziz Abdulrahman Al-Askar

**Affiliations:** Botany and Microbiology Department, College of Science, King Saud University, P.O. 2455, Riyadh, 11451 Saudi Arabia

**Keywords:** Candida vaginitis, Anticandidal bioassay, *Syzygium aromaticum*, GC-MS, Cytotoxicity

## Abstract

**Background:**

Candida vaginitis is a global health hazard that increases morbidity among women of childbearing age. Recent studies have revealed a high incidence of drug-resistant *Candida* strains. Additionally, treating Candida vulvovaginitis during pregnancy is challenging as antifungal therapy is associated with fetal abnormalities. Hence, it is important to develop novel therapeutic strategies to treat vulvovaginal candidiasis.

**Methods:**

In this study, we used the disc diffusion method to evaluate the anticandidal activity of different *Syzygium aromaticum* extracts (methanol, ethyl acetate, n-hexane, and diethyl ether) against *C. albicans*, *C. glabrata,* and *C. tropicalis*. Furthermore, gas chromatography-mass spectrometry (GC-MS) analysis of different *S. aromaticum* extracts was performed to determine active components exhibiting anticandidal activity. Cytotoxicity of different clove extracts against the HUH7 cell line was evaluated.

**Results:**

The ethyl acetate extract exhibited the highest antifungal activity against *C. albicans*, *C. glabrata*, and *C. tropicalis* with inhibition zone diameters of 20.9, 14.9, and 30.7 mm, respectively. The minimum inhibitory concentration of the *S. aromaticum* ethyl acetate extract was 250 μg/disc against *C. tropicalis*, and 500 μg/disc against *C. albicans* and *C. glabrata*, while the minimum fungicidal concentration was 0.5 mg/disc against *C. tropicalis* and 1 mg/disc against the *C. albicans* and *C. glabrata*. GC-MS analysis of the ethyl acetate extract revealed the main bioactive compound as eugenol (58.88%), followed by eugenyl acetate (23.86%), trans-caryophyllene (14.44%), and α-humulene (1.88%). The cytotoxicity assay indicated that the diethyl ether extract demonstrated the lowest toxicological effect against the HUH7 cell line, with a relative IC_50_ of 62.43 μg/ml; the methanolic extract demonstrated a higher toxicity (IC_50_, 24.17 μg/ml).

**Conclusion:**

As the *S. aromaticum* extract exhibited high antifungal activity at low concentrations, it can be a potential source of natural antifungal drugs.

## Background

Candidal vulvovaginitis is a common inflammatory disease among women, caused by an infection of the *Candida* species, especially *C. albicans* [1]. Candida vaginitis is characterized by vaginal discharge, pruritus, itching, dyspareunia, and erythematous vulva [2]. Epidemiological studies have indicated that *C. albicans* (70.0–89.0%) is the main causative agent of Candida vaginitis, followed by *C. glabrata* (3.4–20.0%) [3–6]. The high incidence of infection rates among pregnant women may be attributed to the high secretion of sex hormones during pregnancy [7, 8]. A prescription of antifungal drugs during pregnancy is a challenging task as antifungals are accompanied by possible fetal toxicity and teratogenicity [9]. The virulence factors of the *Candida* species including hyphae formation, production of extracellular enzymes, adhesion, and biofilm formation, help in fungal colonization in the host and the establishment of fungal infection in the vagina [10–13]. *Candida* pathogens adhere to the epithelial cells of the vagina to initiate fungal infection through the production of proteins called adhesins [14]. Moreover, the formation of *C. albicans* pseudomycelium enhances the ability of the fungus to invade the host vaginal tissues [15]. Additionally, *Candid*a species secrete several extracellular enzymes such as lipases, phospholipases, and hemolysins that aid in the adhesion and host tissue invasion [10]. In the USA, *C. glabrata* is the second most common causative agent of invasive candidiasis (12–18%), reportedly exhibiting resistance against fluconazole [16]. An earlier study reported that approximately 94% of all *C. albicans* isolates from vaginitis patients exhibit resistance to fluconazole [17]. Furthermore, another study has reported that certain *Candida* isolates exhibit resistance to fluconazole and econazole [18]. *Syzygium aromaticum* (clove) is an aromatic medicinal plant that belongs to the Myrtaceae family [19]. Reportedly, clove oil possesses antifungal, antibacterial, antiviral, and insecticidal properties due to the presence of phytoactive compounds such as eugenol, eugenyl acetate, and β-caryophyllene [19–21]. The antifungal activity of *S. aromaticum* has been demonstrated by Khan et al. (2009), who reported that the ethanolic extract of clove was highly effective against *C. albicans* with a Minimum inhibitory concentration (MIC) value at 156 μg/mL [22]. The same result has been confirmed by Gonelimali et al. (2018) and Sahal et al. (2019), who detected the potency of clove extracts against *C. albicans* and *C. tropicalis* strains at concentrations of 20% w/v and 500 μl/mL, with inhibition zones diameter of 25.2 and 28 mm, respectively [23, 24]. The high incidence of vaginal candidiasis among pregnant women, in addition to the emergence of resistant *Candida* strains to different antifungal agents, enhances the necessity to formulate novel and safe natural therapeutic agents. Hence, this study aimed to investigate the anticandidal activity of different *S. aromaticum* extracts against three *Candida* species.

## Methods

### Preparation of plant extracts

The extraction procedure was performed using different organic solvents (methanol, ethyl acetate, diethyl ether, and n-hexane), demonstrating different polarities to allow the extraction of all hydrophilic and lipophilic bioactive compounds. The flower buds of *S. aromaticum* were obtained from a local market in Riyadh, Saudi Arabia. We identified the plant material and the identification was confirmed by the Saudi herbarium, Botany Department, College of Science, King Saud University. The plant material was deposited at the herbarium with a voucher number (KSU-14682). Clove buds were disinfected using 0.5% sodium hypochlorite solution (NaOCl), washed three successive times using sterile distilled water, and allowed to dry. The dried plant material was homogeneously powdered using a mechanical mortar. The powdered sample (15 g) was added to 200 mL of the different organic solvents and incubated on a magnetic stirrer for 48 h. Next, the mixture was centrifuged at 9000 rpm for 10 min to remove residues. The supernatant was filtered using a Whatman filter paper to obtain a clear filtrate. The filtrate was concentrated using a rotatory evaporator and stored at 4 °C until use. The yield of the extract was calculated using the following formula:

$$ \mathrm{Percentage}\kern0.17em \mathrm{extract}\kern0.17em \mathrm{yield}=\left(\mathrm{R}/\mathrm{S}\right)\times 100 $$

where R is the weight of the residue of the plant extract; S is the raw plant sample weight.

### Candida isolates

Three *Candida* species, *C. albicans*, *C. tropicalis*, and *C. glabrata* were obtained from King Khalid Hospital, Riyadh, Saudi Arabia. The fresh inoculum was prepared by subculturing each *Candida* species onto Sabouraud dextrose agar (SDA) medium at 35 °C for 48 h.

### Preparation of candidal inoculum

SDA slants were prepared and inoculated with different *Candida* species. The fungus was harvested using 5 mL sterile saline solution. The absorbance of the fungal suspension was measured at 560 nm using a spectrophotometer and the cell count was adjusted to attain a viable cell count of 10^7^ CFU/mL for each *Candida* species.

### Anticandidal bioassay

The anticandidal activity of different *S. aromaticum* extracts was evaluated by the disc diffusion method. The anticandidal bioassay was performed to evaluate the antifungal potency of the extract, in which 10 mL SDA medium was poured into the sterile petri dishes as a basal layer, followed by the addition of 15 mL seeded medium previously inoculated with the prepared microbial suspension (1 mL of fungal suspension/100 mL of medium) to attain a viable cell count of 10^5^ CFU/mL. The sterilized filter paper discs (diameter 8 mm) were loaded with 10 mg of different clove extracts and placed over the seeded plates after solidification [25, 26]. Terbinafine was used as a positive control at a concentration of 50 μg/disk according to CLSI guidelines. Interpretation criteria of terbinafine as the antifungal agent corresponding to the inhibition zone diameter was as follows: ≥ 20 mm, sensitive; 12–19 mm, dose-dependent; ≤ 11 mm, resistance [27]. Eugenol (Sigma-Aldrich, USA), the main active constituent of clove, was also used as a standard phytoactive compound. Sterile filter paper discs loaded with 50 μg of eugenol were placed over the seeded plates [28]. The plates were incubated at 4 °C for 2 h to allow the diffusion of the clove extracts throughout the medium. The plates were further incubated at 35 °C for 48 h and the inhibition zone diameter was measured using a Vernier caliper as an indication of antifungal activity.

### Determination of minimum inhibitory concentration (MIC)

MIC was defined as the lowest concentration of plant extract exhibiting antifungal activity. MIC was evaluated only for the ethyl acetate extract of *S. aromaticum* as the most effective clove extract. SDA medium (15 mL) was poured into a sterile petri dish as a basal layer, followed by the addition of 10 mL seeded medium (as described above), and allowed to solidify. Sterile filter paper discs (diameter 8 mm) loaded with different concentrations of *S. aromaticum* ethyl acetate extract (0.125, 0.25, 0.50, 1.0, 2.0 and 4.0 mg/disc) were placed over the seeded medium. The plates were incubated at 5 °C to allow extract diffusion. The plates were then incubated at 35 °C for 48 h and the inhibition zone diameter was measured using a Vernier caliper. The lowest concentration that exhibited antifungal activity against *Candida* species was recorded as the MIC.

### Determination of minimum fungicidal concentration

Minimum fungicidal concentration (MFC) was defined as the lowest concentration of the clove extract exhibiting fungicidal activity. Streaks obtained from the inhibition zone area of MIC and two successive concentrations were cultured on freshly prepared SDA plates. The plates were incubated at 35 °C for 48 h and the lowest concentration that exhibited no fungal growth was recorded as MFC.

### Gas chromatography-mass spectrometry (GC-MS) analysis of *S. aromaticum* extracts

Phytochemical analysis of the *S. aromaticum* extracts was performed by GC-MS for the detection of active compounds exhibiting antifungal activity. The GC-MS analysis was performed using the GC-MS Thermo Trace GC Ultra / TSQ Quantum GC. The phytochemical analysis was performed using a TR5-MS capillary column, (30 m × 0.25 mm; 0.25 μm film thickness). The oven was programmed to a ramp rate of 6 °C/min to increase the temperature from 40 to 200 °C. The operating conditions were as follows: helium as a carrier gas with a flow rate of 1 mL/min, injector and detector temperatures were 250 °C, split ratio was 1:50. The conditions for mass spectrometry were as follows: mass range from m/z, 40–400 amu; ionization potential 70 eV; electron multiplier energy 2000 V. The chemical constituents of the clove bud extracts were identified by comparing the results of the GC-MS analysis with the reference retention time and spectral mass data of the NIST database.

### Cytotoxicity assay

The human hepatoma (HUH7) cell line was obtained from the Zoology department, College of Science, King Saud University, Saudi Arabia. The toxicity of different clove extracts of diethyl ether, ethyl acetate, methanol, and n-hexane against the HUH7 cell line was evaluated using the 3-(4,5-dimethylthiazol)-2,5-diphenyl tetrazolium bromide (MTT) assay [29]. The cells were cultured in minimal essential medium (Sigma-Aldrich, USA) supplemented with 0.1% gentamicin (Virbac) and 5% fetal calf serum (Adcock-Ingram), incubated in a 5% CO_2_ incubator. HUH7 cells were inoculated in 96-well plates and incubated at 37 °C for 24 h in an 5% CO_2_ incubator for cell adherence to the plate. The crude clove extracts were redissolved in methanol (10 mg/mL), and appropriate dilutions were prepared. Cells were treated with the extracts of concentrations ranging from 0.0065 to 1 mg/ml. After treatment for 48 h, the supernatant was removed and the developing solution (MTT) was added at a concentration of 5 mg/mL to the wells for the formation of formazan crystals. The plates were incubated at 37 °C for 4 h and supernatants were removed. Finally, 50 μL of dimethyl sulfoxide (DMSO) was added to the wells, stabilizing the formed formazan crystals. Absorbance of the soluble formazan in plates was measured at a wavelength of 570 nm. The absorbance corresponding to the concentration inducing a 50% inhibition of cell viability (IC_50_) was calculated.

### Statistical analysis

The susceptibility of *Candida* to different clove extracts was analyzed with GraphPad Prism 5.0 (GraphPad Software, Inc., La Jolla, CA, USA) using one-way analysis of variance and Tukey’s test. The data are presented as mean ± standard error for triplicates. The data were considered statistically significant when the *P*-value was less than 0.05.

## Results

### Extract yield

The highest *S. aromaticum* extract yield (6.09%) was obtained using diethyl ether, followed by methanol (4.67%), n-hexane (3.02%), and ethyl acetate (2.57%).

### Anticandidal activity

Screening the antifungal activity of different *S. aromaticum* extracts against *Candida* species was performed to evaluate the most effective solvent for extraction of active ingredients. All clove extracts exhibited antifungal activity against the different *Candida* strains, with different susceptibility patterns. The ethyl acetate extract of *S. aromaticum* was the most effective extract, demonstrating a high antifungal efficiency against *C. albicans*, *C. glabrata,* and *C. tropicalis*, with inhibition zone diameters of 20.9, 14.9, and 30.7 mm, respectively. The methanolic clove extract of exhibited antifungal potency against all tested *Candida* strains, with inhibition zone diameters of 16.03, 12.93, and 25.6 mm. The inhibition zone diameters of the diethyl ether extract were 19.43, 15.93, and 23.7 mm, respectively (Table 1). *C. albicans* and *C. tropicalis* were sensitive, while *C. glabrata* was more resistant to the antifungal agent terbinafine with inhibition zone diameters of 21.5, 24.33, and 9.6 mm, respectively. In addition, eugenol was used as a standard component of *S. aromaticum* extracts, exhibiting antifungal potency against all evaluated (*C. albicans*, *C. glabrata,* and *C. tropicalis*) strains, with inhibition zone diameters of 15.67, 11.21, and 19.93 mm, respectively. The ethyl acetate clove extract was highly effective compared with the control (terbinafine), indicating a high efficiency against *C. glabrata* and *C. tropicalis*, significantly higher (*P* < 0.05) than the control.

**Table 1:** Antimicrobial activity of different *S. aromaticum* extracts against *Candida* strains

*S. aromaticum* extracts (10 mg/ disc)	Inhibition zone diameter (mm) of *Candida* strains		
	*C. albicans*	*C. glabrata*	*C. tropicalis*
Diethyl ether extract	19.43 ± 0.15	15.93 ± 0.15	23.70 ± 0.17
Ethyl acetate extract	20.93 ± 0.32	14.90 ± 0.21	30.77 ± 0.95
Methanolic extract	16.03 ± 0.83	12.93 ± 0.20	25.60 ± 1.16
N-hexane extract	18.93 ± 0.03	14.07 ± 0.09	30.37 ± 0.26
Control (50 μg/disc)	21.50 ± 0.06	9.60 ± 0.06	24.33 ± 0.13
Eugenol	15.67 ± 0.34	11.21 ± 0.24	19.93 ± 0.42

### MIC and MFC of *S. aromaticum* ethyl acetate extract

**Fig. 1:**
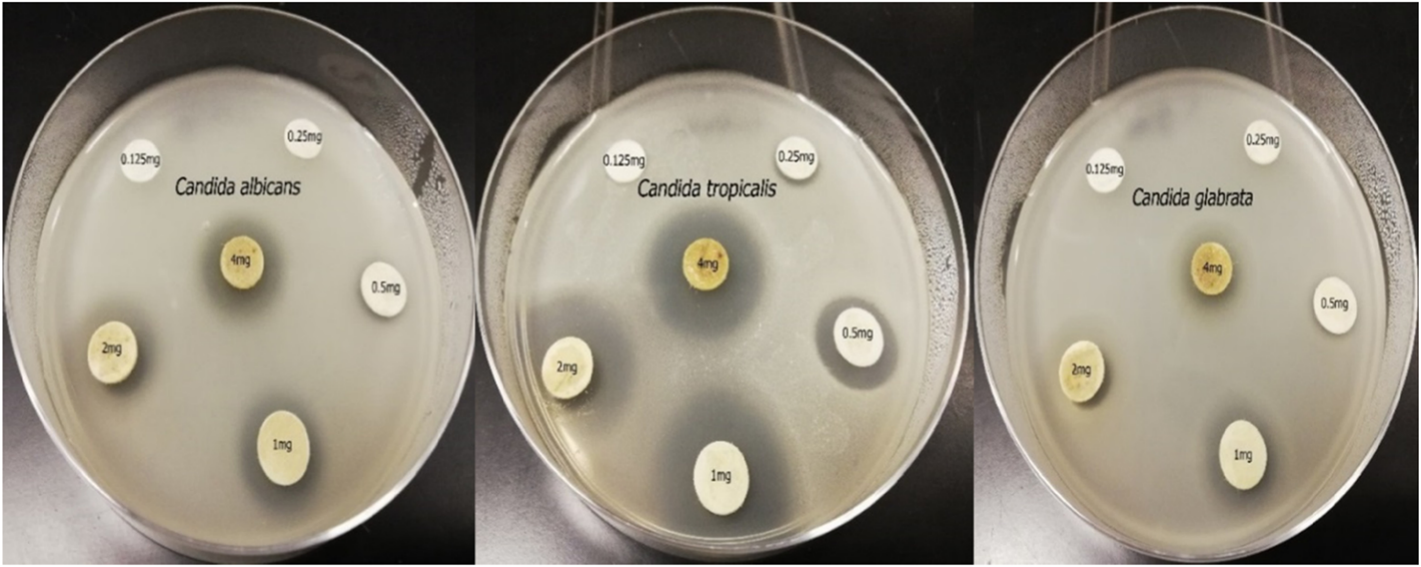
Minimum inhibitory concentration of *S. aromaticum* ethyl acetate extract against *Candida* species

The MIC of *S. aromaticum* ethyl acetate extract against *C. tropicalis* was 0.25 mg/disc, with an inhibition zone diameter of 8.4 mm. The MIC against *C. albicans* and *C. glabrata* was 0.5 mg/disc, with inhibition zone diameters of 8.83 and 8.07 mm, respectively (Table 2). The MIC of *S. aromaticum* ethyl acetate extract against *C. tropicalis* was lower than that against both *C. albicans* and *C. glabrata*. Hence, *C. tropicalis* the most sensitive to this clove extract (Fig. 1). The MFC of the *S. aromaticum* extract against *C. tropicalis* was 0.5 mg/disc as no growth of *Candida* was observed at this concentration. Similarly, no *C. albicans* and *C. glabrata* growth was observed at 1 mg/disc.

**Table 2:** Minimum inhibitory concentrations of *S. aromaticum* ethyl acetate extract against pathogenic *Candida* strains

Concentration of extract (mg/disc)	Inhibition zone diameter (mm) of *Candida* strains		
	*C. albicans*	*C. glabrata*	*C. tropicalis*
0.125	0.0 ± 0.0	0.0 ± 0.0	0.0 ± 0.0
0.250	0.0 ± 0.0	0.0 ± 0.0	8.40 ± 0.06
0.500	8.83 ± 0.16	8.07 ± 0.09	13.40 ± 0.17
1.000	13.77 ± 0.72	11.30 ± 0.12	20.73 ± 0.72
2.000	15.73 ± 0.03	12.60 ± 0.06	23.10 ± 0.17
4.000	16.83 ± 0.32	13.83 ± 0.38	24.17 ± 0.20

### Gas chromatography-mass spectrometry analysis (GC-MS) of *S. aromaticum* extracts

**Fig. 2:**
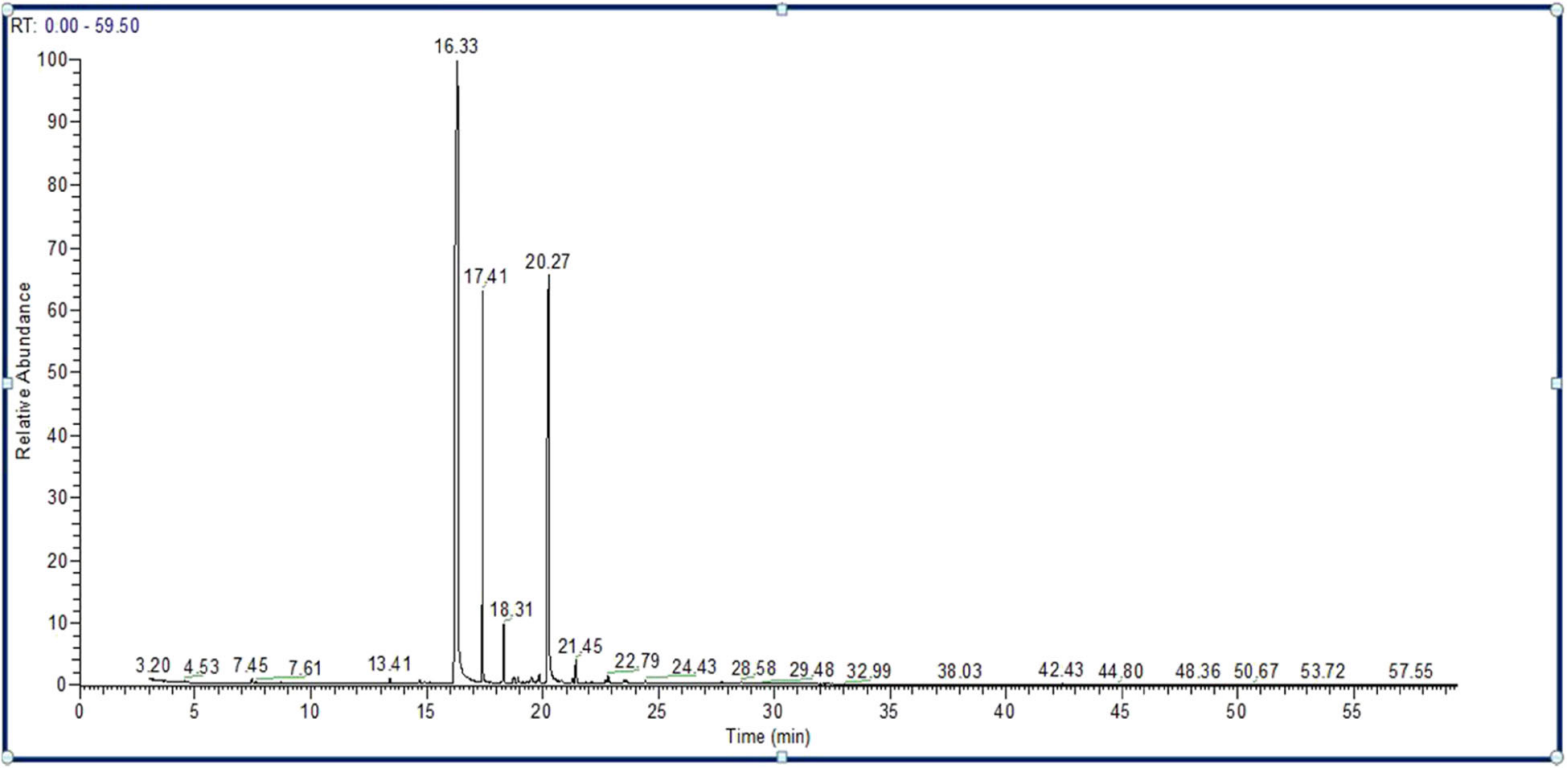
Chromatogram of *S. aromaticum* ethyl acetate extract

**Fig. 3:**
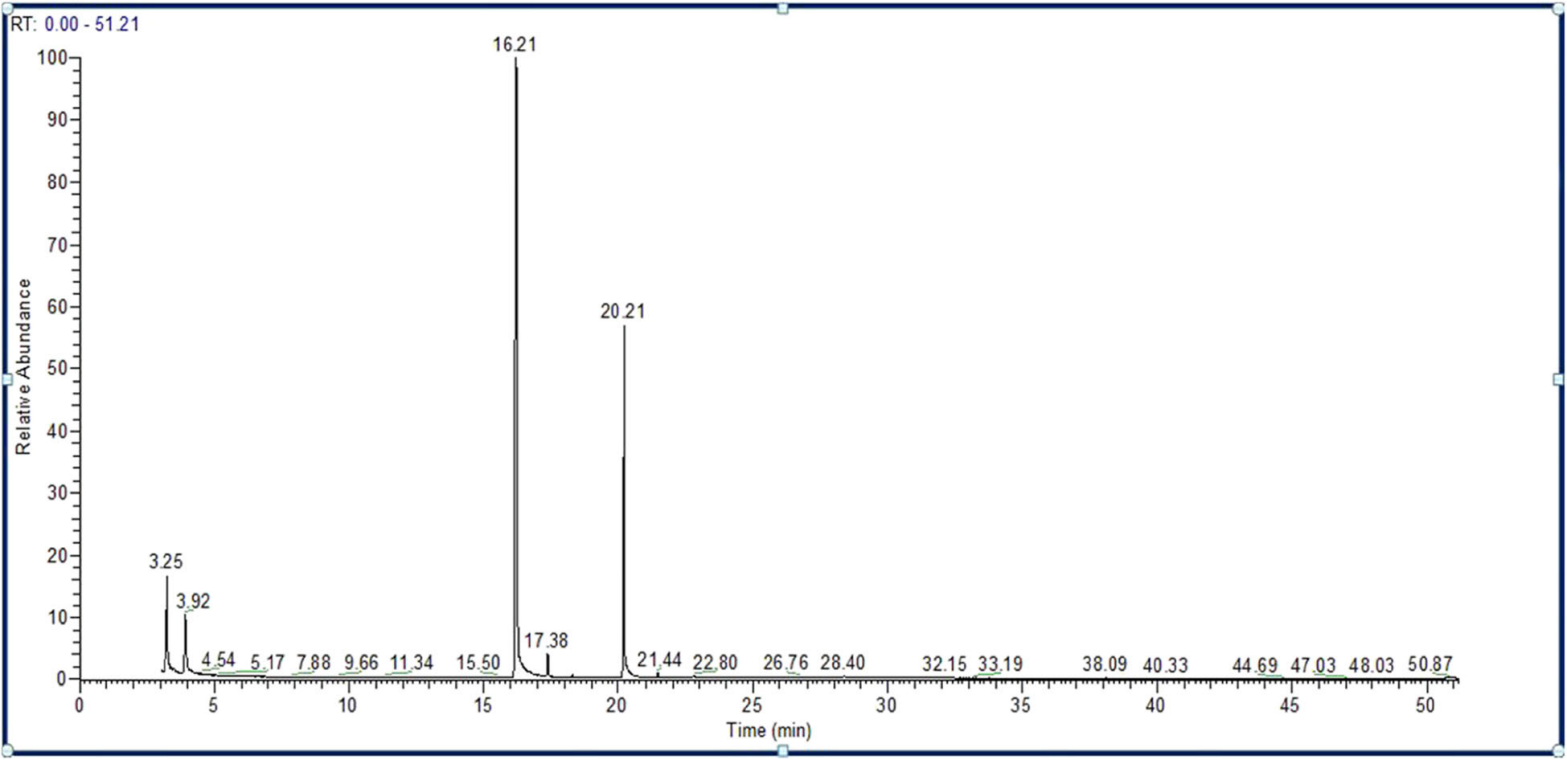
Chromatogram of *S. aromaticum* diethyl ether extract

**Fig. 4:**
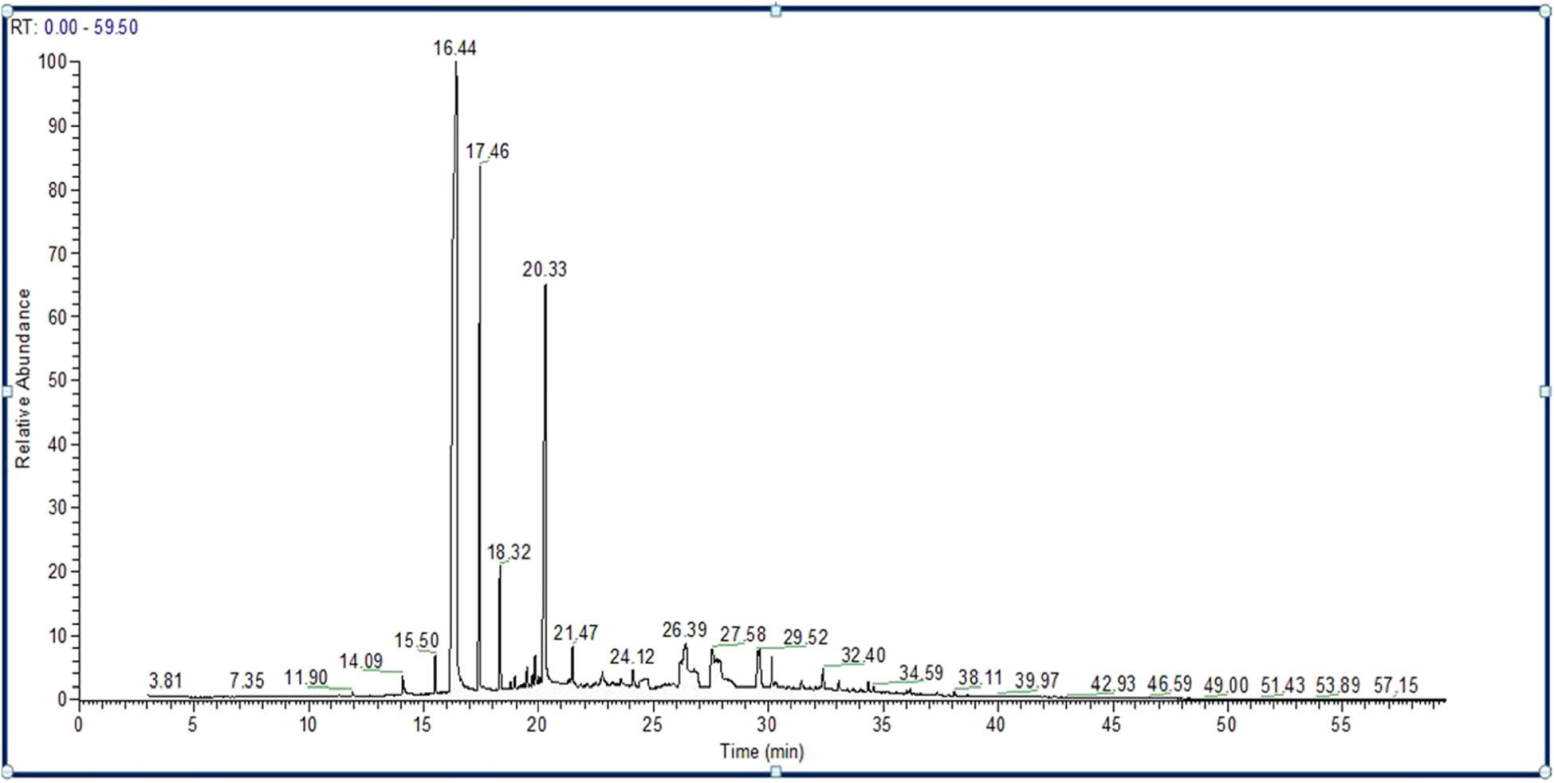
Chromatogram of *S. aromaticum* methanolic extract

**Fig. 5:**
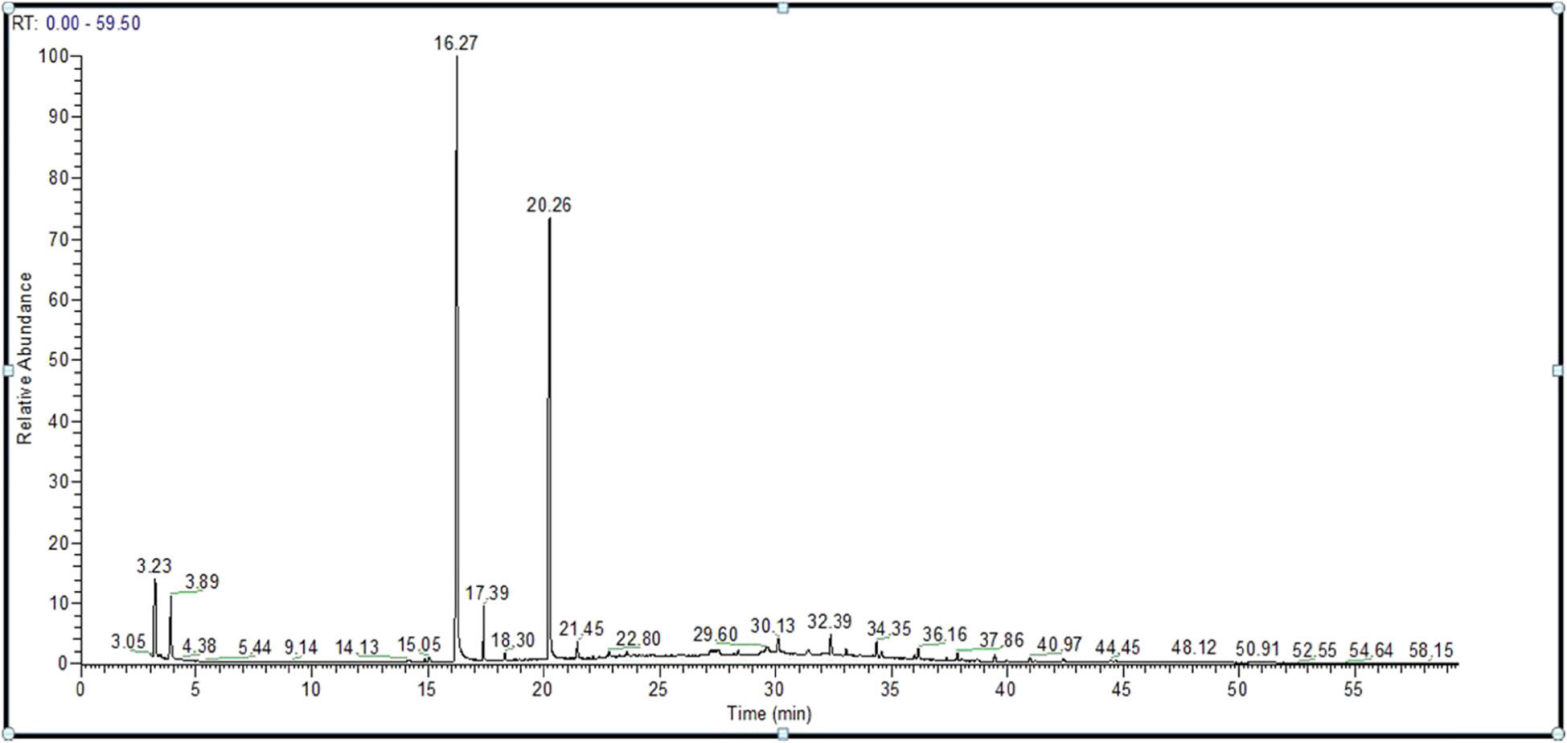
Chromatogram of *S. aromaticum* n-hexane extract

GC-MS analysis of *S. aromaticum* ethyl acetate extract revealed that eugenol (58.88%) was the most abundant active component, followed by eugenyl acetate (23.86%), trans-caryophyllene (14.44%), α-humulene (1.88%), caryophyllene oxide (0.74%), and longipinocarvone (0.19%) (Table 3 and Fig. 2). Furthermore, the main bioactive component of the diethyl ether extract was eugenol (66.48%), followed by eugenyl acetate (31.73%), trans-caryophyllene (1.63%), caryophyllene oxide (0.31%), and α-humulene (0.21%) (Table 4 and Fig. 3). In contrast, new phytochemical constituents detected in the *S. aromaticum* methanolic extract included musk ketone (2.78%), α-cubebene (0.79%), chavicol (0.78%), β-cadinene (0.59%), and α-farnesene (0.37%). Other constituents such as eugenol (55.58%), eugenyl acetate (19.83%), trans-caryophyllene (15.71%), α-humulene (2.46%), and caryophyllene oxide (1.10%) were also detected (Table 5 and Fig. 4). Similarly, eugenol (61.37%) was the most abundant bioactive component in the n-hexane extract (Table 6 and Fig. 5).

**Table 3:** Phytochemical components of *S. aromaticum* ethyl acetate extract

Compounds	Chemical formula	M.W.	Retention time (min.)	% of Total
Eugenol	C_10_H_12_O_2_	164	16.33	58.88
trans-Caryophyllene	C_15_H_24_	204	17.41	14.44
α-Humulene	C_15_H_24_	204	18.31	1.88
Eugenol acetate	C_12_H_14_O_3_	206	20.27	23.86
Longipinocarvone	C_15_H_22_O	218	21.30	0.19
Caryophyllene oxide	C_15_H_24_O	220	21.45	0.74

*M.W.* Molecular weight

**Table 4:** Phytochemical components of *S. aromaticum* diethyl ether extract

Compounds	Chemical formula	M.W.	Retention time (min.)	% of Total
Eugenol	C_10_H_12_O_2_	164	16.21	66.48
trans-Caryophyllene	C_15_H_24_	204	17.38	1.63
α-Humulene	C_15_H_24_	204	18.29	0.21
Eugenol acetate	C_12_H_14_O_3_	206	20.21	31.37
Caryophyllene oxide	C_15_H_24_O	220	21.44	0.31

*M.W.* Molecular weight

**Table 5:** Phytochemical components of *S. aromaticum* methanolic extract

Compounds	Chemical formula	M.W.	Retention time (min)	% of total
Chavicol	C_9_H_10_O	134	14.09	0.78
α-Cubebene	C_15_H_24_	204	15.50	0.79
Eugenol	C_10_H_12_O_2_	164	16.44	55.58
trans-Caryophyllene	C_15_H_24_	204	17.46	15.71
α-Humulene	C_15_H_24_	204	18.32	2.46
α-Farnesene	C_15_H_24_	204	19.50	0.37
β-Cadinene	C_15_H_24_	204	19.84	0.59
Eugenol acetate	C_12_H_14_O_3_	206	20.33	19.83
Caryophyllene oxide	C_15_H_24_O	220	21.47	1.10
Musk ketone	C_14_H_18_N_2_O_5_	294	29.52	2.78

*M.W.* Molecular weight

**Table 6:** Phytochemical components of *S. aromaticum* n-hexane extract

Compounds	Chemical formula	M.W.	Retention time (min.)	% of Total
Eugenol	C_10_H_12_O_2_	164	16.27	61.37
trans-Caryophyllene	C_15_H_24_	204	17.39	0.52
α-Humulene	C_15_H_24_	204	18.30	0.34
Eugenol acetate	C_12_H_14_O_3_	206	20.26	34.95
Caryophyllene oxide	C_15_H_24_O	220	21.45	0.82

*M.W.* Molecular weight

### Cytotoxicity assay

**Fig. 6:**
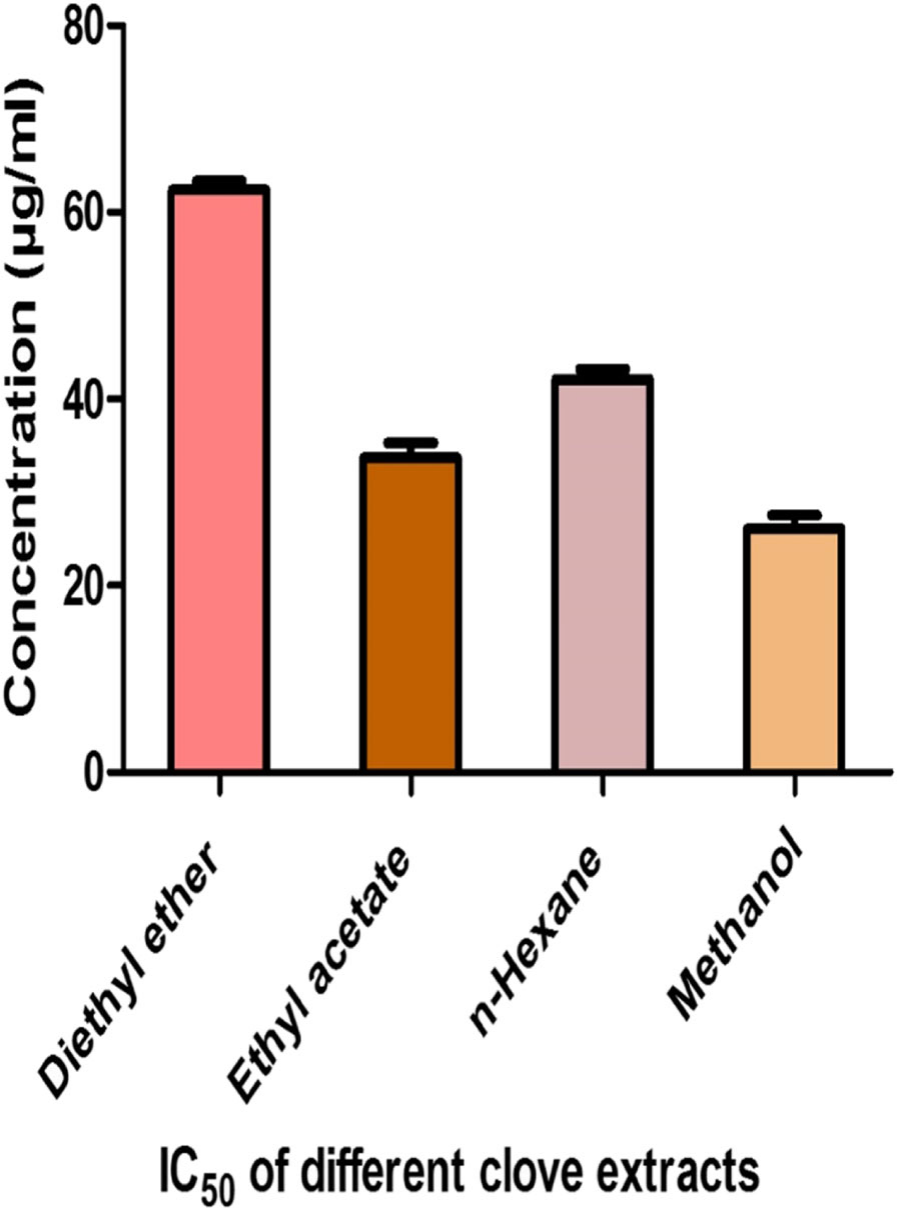
IC_50_ of different clove extracts against HUH7 cell line

The diethyl ether clove extract demonstrated the lowest toxic effect against the HUH7 cell line, with a relative IC_50_ of 62.43 μg/ml, while higher toxicity of was detected with the methanolic extract, with an IC_50_ of 24.17 μg/ml. Moreover, the n-hexane and ethyl acetate extracts of clove exhibited moderate toxicity against HUH7 cells with relative IC_50_ values of 42.19 and 33.68, respectively (Fig. 6).

## Discussion

All extracts of *S. aromaticum* exhibited antifungal activity against the concerned candidal strains evaluated in this study (Table 1). These results concurred with those of Mansourian et al., demonstrating that *S. aromaticum* extracts (10 mg/100 μl) possessed antifungal activity against *C. albicans*, with an inhibition zone diameter of 29.6 mm [25]. Reportedly, clove extracts possess antioxidant and antimicrobial properties due to the presence of phenolic compounds, such as flavonoids, hydroxybenzoic acids, and hydroxyphenyl propenes [30]. Phytochemical analysis of the *S. aromaticum* ethyl acetate extract demonstrated that eugenol was the most abundant active component (58.88%), followed by eugenyl acetate (23.86%), trans-caryophyllene (14.44%), and α-humulene (1.88%). These phytochemicals were consistent with the study by Jirovetz et al., who reported that eugenol was the most abundant active phytochemical component, constituting 76.8% of the total active compound [31]. Additionally, similar results were reported by Chaieb et al., demonstrating that clove oil was predominantly composed of eugenol (70%), followed by eugenyl acetate (5.6%), and β-caryophyllene (1.4%) [32]. The high efficiency of the ethyl acetate clove extract as an antifungal agent offers a potential natural anticandidal drug for the treatment of *Candida* vaginitis, avoiding the side effects associated with several chemotherapeutic agents used in the treatment of vaginitis. In addition, Chami et al. ascertained that clove extract constituents are a promising source for the curative and prophylactic therapy of vulvovaginal candidiasis [33]. The inhibition zone diameters recorded with eugenol (50 μg) as a standard compound against the concerned *Candida* species were less than those recorded for the crude clove extract (10 mg). These results may be attributed to the high content of eugenol in different extracts, confirmed by GC-MS analysis of clove. GC-MS analysis of *S. aromaticum* extracts (diethyl ether, ethyl acetate, n-hexane, and methanolic) revealed that eugenol was the main active compound, with relative percentages of 66.48, 58.88, 55.58, and 61.37%, respectively.

Eugenol exhibited antifungal potency against *C. albicans* strains, demonstrating inhibition zone diameters of 15.67 mm. This result was consistent with that of Pavesi et al., who detected the potency of eugenol at a concentration of 57 μg/disk against *C. albicans*, with an inhibition zone diameter of 12.1 mm [28]. In the present study, the ethyl acetate clove extract demonstrated the highest antifungal efficacy against the different *Candida* species compared to the other solvent extracts evaluated. The antifungal potency of *S. aromaticum* could be attributed to the high eugenol content of clove extracts, which inhibits the biosynthesis of ergosterol, a component of the microbial cell membranes. These may lead to the disruption of microbial cell membrane permeability causing cell death [34]. Other researchers have attributed the antifungal activity to eugenyl acetate which inhibits germ tube formation, prevents the formation of candidal biofilms, and enhances phagocytic activity of macrophages against the *C. albicans* species [35]. The current study revealed that *S. aromaticum* extracts exhibited strong antifungal potency against *Candida* species at low concentrations (250 μg/disc). Hence, it could be used as a potential source of natural antifungal drugs. The cytotoxicity assay confirmed that the diethyl ether clove extract possessed the lowest toxicity, while the ethyl acetate extract exhibited moderate toxicity against the HUH7 cancer cell lines, with IC_50_ values of 62.43 and 33.68 μg/ml respectively. These results were in accordance with that of Kumar et al. who have reported clove oil toxicity against MCF-7 human breast cancer cell lines, with an IC_50_ value of 36.43 μg/ml [36]. Vijayasteltar et al. have demonstrated the safety of clove bud extracts as dietary supplements in Wistar rats, confirming the absence of toxicological changes in behavioral observations, body weights, organ weights, ophthalmic examinations, feed consumption, hematology, urinalysis, and clinical biochemistry parameters compared to the untreated group of animals [37].

## Conclusion

*Syzygium aromaticum* extracts exhibited a highly antifungal efficiency against the most common and predominant strains causing candidal vaginitis. Ethyl acetate was the most effective organic solvent in the extraction process, producing a high yield of clove active constituents. Furthermore, the results demonstrated a high potency of the clove extract compared with terbinafine (control). Hence, it could be used as a natural, safe, and effective antifungal agents. Moreover, it could be prescribed as a substitute to several chemotherapeutic agents used in the candidal vaginitis therapy for external use, eliminating the extensive side effects associated with these chemical agents, especially in pregnant women.

## Data Availability

The datasets used and/or analyzed during the current study is available from the corresponding author on reasonable request.

## Abbreviations

CFU: Colony forming unit
GC-MS: Gas Chromatography-Mass Spectrometry
HUH7: Human hepatoma cell line
IC_50_: The half maximal inhibitory concentration
MFC: Minimum fungicidal concentration
MIC: Minimum inhibitory concentration
MTT: 3-(4,5-dimethylthiazol)-2,5-diphenyl tetrazolium bromide
SDA: Sabouraud dextrose agar
